# Praxis function in the ADL Observation Scale: Spanish cross-cultural adaptation and psychometric properties

**DOI:** 10.1371/journal.pone.0341856

**Published:** 2026-01-30

**Authors:** Laura Sánchez-Bermejo, Pedro Jesús Milla-Ortega, José Manuel Pérez-Mármol

**Affiliations:** 1 Instituto de Investigación Biosanitaria ibs.GRANADA, Granada, Spain; 2 Department of Physiatry and Nursing, Faculty of Health Sciences, University of Zaragoza, Zaragoza, Spain; 3 Emergencies Primary Care Service, Granada Health District, Granada, Spain; 4 Department of Physiotherapy, Faculty of Health Sciences, University of Granada, Granada, Spain; PLOS: Public Library of Science, UNITED KINGDOM OF GREAT BRITAIN AND NORTHERN IRELAND

## Abstract

**Objective:**

To cross-cultural adapt the ADL Observation Scale and evaluate its psychometric properties in a sample of Spanish-speaking patients with stroke.

**Methods:**

A validation study that included cross-cultural adaptation, structural validity assessment, reliability, and diagnostic accuracy analysis. The study followed COSMIN and STARD criteria. Translation and cross-cultural adaptation of the ADL Observation Scale followed a backward translation process. Structural validity was assessed with confirmatory factor analysis, and internal consistency was analyzed using Cronbach’s alpha and McDonald’s omega coefficient. Cut-off points were estimated using ROC analysis, and diagnostic accuracy was evaluated using sensitivity, specificity, and positive and negative predictive values. Internal consistency, cut-off points, and diagnostic accuracy were calculated for two different age groups (≥65 years; > 65 years).

**Results:**

The Spanish version of the ADL Observation Scale was obtained after cross-cultural adaptation. The psychometric properties were tested in a sample of 180 patients with stroke. Structural validity showed a four-factor structure of the scale (RMSEA = 0.078; CFI = 0.952; TLI = 0.934). Internal consistency for each factor indicated Omega values between 0.641, 95% CI [0.495, 0.753], and 0.915, 95% CI [0.882, 0.936]. Cut-off points, also calculated for each factor, ranged from ≥1 to ≥4 points. Sensitivity, specificity, and positive and negative predictive values showed high values, indicating the scale’s discriminatory capacity for correctly classifying patients with praxis deficits.

**Conclusion:**

The ADL Observation Scale has a four-factor structure, and it is a reliable instrument for evaluating the levels of praxis function in daily living among Spanish-speaking patients with stroke. The scale also has adequate diagnostic accuracy in detecting patients with praxis deficits.

## Introduction

Upper limb apraxia (ULA) is a cognitive deficit that impairs the ability to plan or perform purposeful movements with the hands or arms [1]. ULA is frequently underdiagnosed, partly because it can go unnoticed in everyday situations and partly due to the lack of appropriate instruments to evaluate praxis function, especially in non-English-speaking contexts [2,3]. Most tools evaluate praxis execution using isolated actions or tasks, such as gesture imitation or pantomime performance. While this evaluation framework contributes to understanding praxis deficits from a neuropsychological perspective, this approach does not capture how individuals interact with their real physical environment in everyday situations. For this reason, the evaluation of praxis function should also include the difficulties experienced by patients while performing activities of daily living (ADL) [4]. This evaluation framework may offer several advantages, as it adopts a holistic and ecological perspective by accounting for all potentially altered components of praxis and being applied in the individual’s natural environment [5].

Within this framework, the ADL Observation Scale is an instrument that evaluates the level of praxis functioning in the performance of daily life activities [6]. This scale may identify apraxia deficits based on the observation of four factors of praxis function: independence, initiation, execution, and control. The independence factor aims to evaluate the level of functional autonomy demonstrated during task performance. This factor is specifically related to the ability to perform tasks without assistance and to implement learned movements in various situations and environments —that is, the transfer of these movements to new contexts [7–9]. The initiation factor indicates the level of gesture planning skills —a dimension recognized in cognitive models of apraxia— and refers to whether the patient begins the action without delay or the need for external assistance. This factor is related to action planning and conceptualization (i.e., ideational apraxia according to the classical models). Initiation comprises various praxis components such as motor plan construction, selection of appropriate tools and objects, and action sequencing— that is, the ability to generate the sequence of movements needed for a task initiation [10–12]. The execution factor is observed through the kinematic organization of movement during ADL execution, including spatial aspects in movement execution (spatial accuracy in gestures), temporal aspects (timing and fluidity of movement), and limb orientation and configuration (positioning of arms, hands, or fingers correctly during upper limb movements) [13,14]. The control factor refers to the executive control of action and reflects the patient’s capacity to monitor their movements, identify potential mistakes, and implement corrections as needed. Control relies on the real-time detection of movement errors and the use of internal and external feedback—such as tactile information or visual cues—to recognize and make movement adjustments [15,16].

The ADL Observation Scale offers a unique approach to evaluating ULA compared to other instruments, such as the TULIA Apraxia test or the Florida Apraxia Battery. On one hand, the TULIA Apraxia test evaluates the presence or absence of ULA through imitation and pantomime tasks, including symbolic, non-symbolic, transitive, and intransitive gestures [17]. Similar to its screening version, the AST TULIA [18]. On the other hand, the Florida Apraxia Battery focuses on the recognition and expression of pantomime and semantic action knowledge [19]. These types of instruments register different dimensions of ULA. However, they evaluate the praxis function in clinical environments without incorporating real objects or the performance of daily activities. In contrast, the ADL Observation Scale provides an ecological evaluation by observing how patients interact with real objects within an everyday context. This perspective is particularly useful in providing information about how ULA manifests itself in real-life scenarios and during the performance of everyday activities [6,20]. Thus, the analysis of the psychometric properties of the ADL Observation Scale would allow clinicians and researchers to recognize the functional implications of ULA across different populations, providing valuable insight into praxis functioning in the performance of daily life activities.

The ADL Observation Scale has been used to assess the effects of living in care units on ULA in people with dementia [21] and post-stroke [22], or to track the progression of ULA and its effect on daily functioning over time [23]. However, despite its utility, the ADL Observation Scale has not yet been validated for use with Spanish-speaking patients. This underscores the need for its cross-cultural adaptation and analysis of its psychometric properties. This analysis should provide information on the scale’s structural validity and internal consistency. Additionally, determining cut-off points and diagnostic accuracy could support the use of the scale in identifying ULA in patients with a high probability of having this syndrome [22]. For these reasons, this study aims to cross-culturally adapt the ADL Observation Scale and evaluate its psychometric properties in a sample of Spanish-speaking patients with stroke.

## Materials and methods

### Study design

This validation study followed different methodological frameworks to ensure the quality of the entire process. COSMIN guidelines were employed to standardize terminology, guide the selection of appropriate psychometric analyses, and structure the methods and results report [24–27]. The translation and cross-cultural adaptation of the ADL Observation Scale followed a forward and backward process, in line with COSMIN recommendations and the most recent evidence [28–30]. Finally, the Standards for Reporting of Diagnostic Accuracy Studies (STARD) guidelines were applied to ensure transparency of diagnostic accuracy analysis [31].

### Sample selection criteria

Participants were recruited from randomly selected public primary care centers in the province of Granada, Spain, from 2022 to 2024. Within each selected center, consecutive sampling was employed to select patients with stroke. Patients potentially eligible for the study were identified by health professionals and informed about the study. Those who met the inclusion criteria and expressed interest were contacted by the research team to confirm their eligibility, provide a more detailed explanation of the study, and schedule the assessment session. The inclusion criteria were: i) a confirmed stroke diagnosis, ii) mild to moderate stroke sequelae, assessed by the National Institutes of Health Stroke Scale (NIHSS), iii) age 18 years or older, and iv) fluency in Spanish. The exclusion criteria included: i) a diagnosis of brain damage unrelated to vascular causes, ii) moderate to severe cognitive impairment, according to the records from clinical history [32], iii) a diagnosis of severe intellectual disability, iv) a diagnosis of severe mental disorder, v) musculoskeletal disorder, vi) peripheral nervous system injuries, and vii) uncorrected sensory impairments.

### Sample size estimation

The sample size was estimated based on the requirements for conducting a confirmatory factor analysis (CFA) [33]. The parameters used were an expected root mean square error of approximation (RMSEA) value of 0.05 [34], an expected comparative fit index (CFI) of 0.95 [35], an average factor loading of 0.70, and an average factor correlation of 0.50 [36] for the multidimensional model composed of four factors and 16 items. The significance level was set at 0.05 (two-tailed), and the statistical power (1-*β*) at 85%. The final calculated sample size for the study resulted in 181 participants.

### Description of the participants

Participants were described using socio-demographic and clinical information. Variables were recorded using an *ad hoc* questionnaire and cross-verified with medical records. The variables registered were age (in years), sex (man, woman), educational level (primary, secondary, vocational, university education), stroke type (ischemic, hemorrhagic, lacunar), affected hemisphere (right, left, both, indeterminate), time since stroke onset (in months), and presence of hemiplegia (absent, present). Sociodemographic and clinical information were summarized and reported using means, frequencies, percentages, and standard deviations as appropriate. Descriptive statistics for the ADL Observation Scale were also summarized using means, standard deviations, minimums, and maximums. SPSS v.28 for Windows was utilized for all descriptive analyses.

### Description of the ADL Observation Scale

The ADL Observation Scale evaluates the levels of praxis function in four daily activities: personal hygiene (washing the face and upper body), dressing (putting on a shirt), feeding (preparing a sandwich), and a fourth activity selected by the evaluator (brushing teeth). The praxis function in these activities is evaluated based on the four specific factors: independence, initiation, execution, and control. Thereby, the scale includes 16 items in total. The independence factor evaluates the ability to complete the praxis actions of the activity autonomously, while the initiation factor evaluates the skills of planning gestures. The execution factor evaluates the ability to execute correctly the praxis actions of the activity in terms of movement execution, temporal aspects, limb orientation, and configuration. Finally, the control factor evaluates the ability to monitor the movements, potential mistakes, and implement corrections as needed. Each factor of praxis function is scored on a scale from zero to three points, with zero indicating adequate praxis ability and no requirement for assistance during the activity. The total score for each factor ranges from zero to 12 points, with higher scores indicating lower levels of praxis performance for each praxis factor. The English ADL Observation Scale exhibits strong internal consistency for the four factors, with a Cronbach’s alpha ranging from 0.79 to 0.81 [6].

### Methodological phases of the study

#### Phase I: Translation and cross-cultural adaptation of the ADL Observation Scale.

Before starting the study, the original authors of the English ADL Observation Scale were informed of the intention to cross-cultural adapt and assess the psychometric properties of this instrument. The translation and cross-cultural adaptation of the scale followed a six-step process. In the first step, two experts with Spanish as their mother tongue independently translated the scale from English to Spanish. One expert was a qualified English translator without knowledge of apraxia, and the second expert was a healthcare professional with knowledge of post-stroke evaluations and apraxia. In the second step, two researchers independently compared the two Spanish translations to identify possible discrepancies in the instructions, items, and scoring methods of the scale. At the same time, a third researcher was selected to address the possible discrepancies and facilitate consensus on meaning, ambiguities, and terminology. This step resulted in a reconciled version of the ADL Observation Scale. In the third step, the reconciled version was independently backward translated into English by a third independent qualified translator, who was blinded to the original scale. In the fourth step, an experts committee (two researchers) reviewed and compared the first two translations, the reconciled version, the backward translated version, and the original scale with two purposes: i) to achieve a consensus about discrepancies and ii) to revise the conceptual, semantic, operational, and item equivalence with the original scale [28,29]. Gender-inclusive language was also analyzed in this step. A pre-final version of the ADL Observation Scale was developed at the end of this step. In the fifth step, the comprehensibility of this pre-final version was assessed in a pilot study with 10 patients with stroke [30]. In the last step, the final version of the Spanish version of the scale was developed, maintaining the premises of clarity, specificity, and precision.

#### Phase II: Assessment of the psychometric properties of the Spanish version of the ADL Observation Scale.

The evaluation of ULA using the ADL Observation Scale lasted approximately 45 minutes, and the sessions were conducted individually in a distraction-free, quiet room with a standard temperature. As recommended by the original authors of the ADL Observation Scale, the evaluator received training sessions before starting the evaluations.

The adequacy of the data for factor analysis was evaluated with Bartlett’s test of sphericity [37] and the Kaiser-Meyer-Olkin analysis (KMO) [38]. A p-value <0.05 for Bartlett’s test was considered satisfactory compatibility of the data, and a KMO value >0.60 was considered acceptable [38]. The psychometric thresholds applied in the study were as follows: for CFA analysis, a ratio of chi-square and degrees of freedom (*χ2/df*) ≤3.000 [39], RMSEA ≤0.080 with a 90% confidence interval [34], CFI ≥ 0.900 [40], Tucker-Lewis index (TLI) ≥0.900 [34], and standardized root-mean-square residual (SRMR) ≤0.050 [41]. Internal consistency was considered satisfactory when Cronbach’s alpha or McDonald’s omega exceeded >0.700 [42]. The AUC was interpreted as no diagnostic discrimination (<0.500), acceptable (0.600 to 0.800), or excellent discrimination (>0.800) [43].

The structural validity of the Spanish version of the ADL Observation Scale was assessed using CFA, a statistical method to evaluate whether observed variables correspond to one or multiple underlying factors. In this line, a four-factor structure of the scale was assumed, based on the four factors of praxis function: independence, initiation, execution, and control [7,11,14,16]. Each factor was composed of the observation of the performance in four ADLs. CFA was run using AMOS version 28 for Windows, encompassing different steps: specification of the model, identification, estimation, assessment, and re-specification if necessary [44]. A maximum likelihood estimator was employed, and to assess the model’s goodness of fit, a path diagram and several indices were considered. To refine and adjust the model, if necessary, the modification indices, along with theoretical considerations, were considered.

Internal consistency of the scale was evaluated for each of the four factors within two separate age groups (≤65 years, > 65 years). The stratification of the sample by age was chosen due to the potential effects of aging-related decline in musculoskeletal and cognitive abilities at this stage of life [45]. Cronbach’s alpha and McDonald’s omega coefficient, both with a 95% confidence interval (CI), were employed. Cronbach’s alpha was estimated using SPSS version 28, while McDonald’s omega coefficient was computed in R software. Test-retest reliability was not evaluated in the present study, and it is recommended that future research include this psychometric property.

Optimal cut-off points for the ADL Observation Scale were calculated for each factor and adjusted based on the same age groups (≤65 years, > 65 years) to improve the diagnostic precision [46,47]. Receiver operating characteristic (ROC) analysis, the area under the curve (AUC) with a 95% confidence interval, [48,49] and the Youden index were employed to identify the optimal cut-off points for each factor. The AST TULIA test [18] served as a binary classifier to dichotomize the sample between individuals with and without ULA. Participants scoring <9 points were classified as having ULA. Both the ADL Observation Scale and the AST TULIA test were administered in the same evaluation session to ensure rigorous comparison and analysis. ROC analysis examined the diagnostic performance of the scale by plotting sensitivity against 1 – specificity at different thresholds [50]. The AUC measured the diagnostic accuracy, and finally, the Youden index was calculated to identify the optimal cut-off point by maximizing the sum of sensitivity and specificity [51]. Microsoft Excel was employed to compute the Youden index, and ROC analysis and AUC calculations were performed using SPSS version 28.

The diagnostic accuracy of the ADL Observation Scale was assessed using four indices, each with its corresponding 95% confidence intervals: sensitivity, specificity, positive predictive value, and negative predictive value. Sensitivity reflects the capacity of the scale to accurately identify individuals with praxis deficits, while specificity indicates how well the scale recognizes those whose praxis function remains unaffected [52]. The positive predictive value represents the probability that individuals identified by the scale as having praxis deficits do indeed experience that deficit. The negative predictive value shows the probability that individuals identified as experiencing praxis deficits truly do not have the deficit [53]. SPSS version 28 was employed to estimate the diagnostic accuracy analysis.

### Ethical considerations

The study received approval from the Ethics Committee for Biomedical Research CEI-Granada in the Province of Granada (Andalusia, Spain) with reference number 1503-N-21. The research process followed the ethical principles outlined in the Declaration of Helsinki, ensuring ethical conduct throughout all stages of the research.

Study participants were informed about their right to participate or withdraw, as well as the confidentiality of their information. Afterwards, all participants signed the written informed consent, retaining a copy for themselves and providing another copy to the researcher.

## Results

### Description of the participants

**Fig 1:**
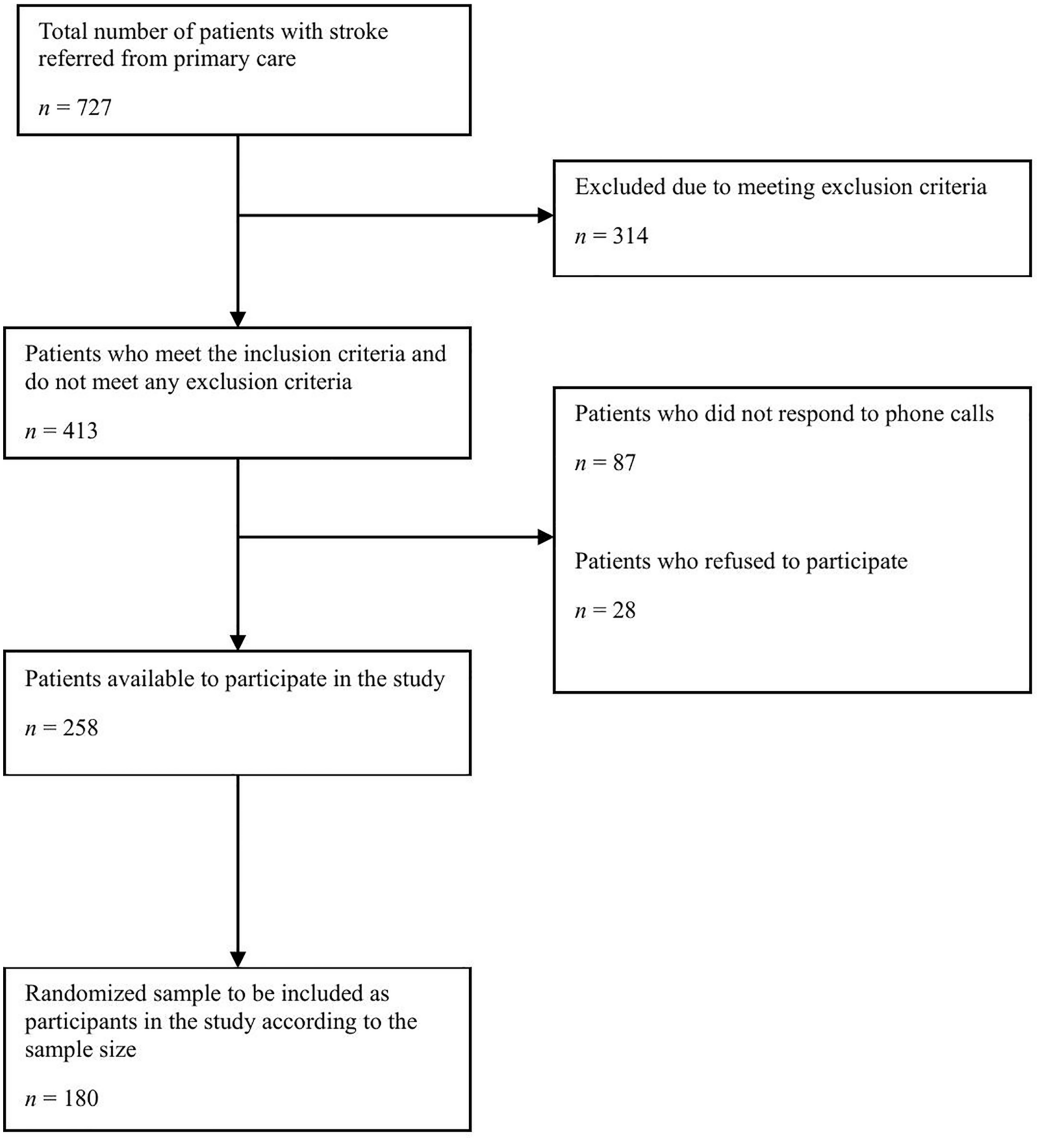
Flowchart illustrating the selection of the study sample.

A total of 413 patients referred from public primary care centers were approached as potential participants to meet the required sample size (n = 180 individuals). The flowchart of the study population is shown in Fig 1.

Sociodemographic and clinical characteristics of the 180 participants of the study are presented in Table 1. Regarding descriptive statistics of the ADL Observation Scale, the mean scores and standard deviations (SD) for the four factors of praxis function were: independence (M = 2.56, SD = 2.87), initiation (M = 0.66, SD = 1.21), execution (M = 2.32, SD = 2.67), and control (M = 0.92, SD = 1.45). The scores ranged from 0 to 10 points.

**Table 1:** Sociodemographic and clinical characteristics of participants.

Sample descriptive data	Participants (N = 180)	
**Socio-demographic data**	**n/ Mean**	**%/ SD**
Age (years)	64.15	11.86
Sex, men	122	67.78
Educational level		
Primary education	71	39.44
Secondary education	51	28.33
Vocational training	28	15.56
University education	30	16.67
**Clinical characteristics**		
Stroke type		
Ischemic stroke	138	76.67
Haemorrhagic stroke	25	13.89
Lacunar stroke	17	9.44
Affected hemisphere		
Right	71	39.44
Left	72	40.00
Both	24	13.33
Indeterminate	13	7.22
Time since stroke onset (months)	29.08	32.40
Presence of hemiplegia	22	12.22

### Phase I: Translation and cross-cultural adaptation

The ADL Observation Scale was translated and cross-cultural adapted without difficulties. Preliminary instructions were added to guide the evaluator before the evaluation. Activity descriptions were made more detailed, following the original authors’ suggestions. In the scoring method, each factor of praxis function was defined, evaluation criteria were linguistically simplified, and, when appropriate, enumerated. The final Spanish version of the ADL Observation Scale is presented in Table 2.

**Table 2:** Spanish version of the ADL Observation Scale.

**ADL Observation Scale**	
Instrucciones preliminares: antes de la llegada de la persona que se va a evaluar, el/la evaluador/a deberá colocar sobre la mesa auxiliar a la mesa principal todo el material necesario para llevar a cabo la evaluación. Una vez que la persona llegue, el/la evaluador/a le explicará de forma clara y sencilla qué se le va a solicitar para la administración de la escala y, cuánto tiempo se va a emplear. Instrucción para dar a la persona evaluada: a lo largo de esta sesión, le voy a pedir que realice cuatro actividades cotidianas. Como ve, en la mesa auxiliar hay varios objetos. Voy a pedirle que elija el/los objetos necesarios para cada actividad, una por una. Tendrá que usar esos objetos como lo haría en la vida real. Intente realizar cada actividad de la forma más precisa posible.	
**Actividades**	
1. Actividad: higiene personal. Lavarse la cara y la parte superior del cuerpo Objeto: esponja Instrucciones para dar a la persona evaluada: haga como si se lavara la cara y la parte superior del cuerpo (hasta la cadera y sin contar la espalda), incluyendo los brazos, tal como lo haría en su rutina diaria de ducha. Recuerde que debe utilizar uno de los objetos que están en la mesa	
2. Actividad: ponerse una camisa Objeto: camisa de botones Instrucciones para la persona evaluada: póngase la camisa que está sobre la mesa, acomódesela y abroche los botones	
3. Actividad: preparar y comer un sándwich Objetos: pan de molde, queso y jamón cocido; alternativa: lechuga, tomate; tenedor y cuchillo Instrucciones para la persona evaluada: prepare un sándwich utilizando los ingredientes que están encima de la mesa. Después, haga como que come un trozo de sándwich utilizando los cubiertos	
4. La cuarta actividad es escogida por el/la evaluador/a, ya sea una actividad relevante para la persona evaluada o una actividad estándar Propuesta de actividad con la que se ha validado el instrumento: Actividad: lavarse los dientes Objetos: cepillo de dientes y pasta de dientes Instrucciones para la persona evaluada: lávese los dientes como lo haría en su rutina diaria, durante al menos un minuto. Utilice los objetos disponibles sobre la mesa para realizar la actividad	
**Parámetros de rendimiento (ejemplos principales) y método de puntaje**	
**Independencia:** capacidad para completar las acciones práxicas de las actividades sin ayuda externa	
0 puntos	La persona realiza las acciones práxicas sin necesidad de ayuda
1 punto	La persona es capaz de realizar las acciones práxicas, pero necesita asistencia verbal, ya sean indicaciones generales o instrucciones detalladas
2 puntos	La persona es capaz de realizar las acciones práxicas, pero necesita cualquier tipo de ayuda física
3 puntos	La persona no puede realizar las acciones práxicas, incluso recibiendo una asistencia completa
**Iniciación:** la capacidad para planificar las acciones práxicas y comenzar el primer paso de cada actividad Higiene personal: llevar la mano hacia la esponja Ponerse una camisa: llevar la mano hacia la camisa Preparar y comer un sándwich: llevar la mano hacia las rebanadas de pan o hacia alguno de los alimentos Lavarse los dientes: llevar la mano hacia el cepillo de dientes o hacia la pasta de dientes	
0 puntos	No se observan problemas, la persona planifica la acción e inicia la actividad
1 punto	Se da al menos uno de los siguientes supuestos: 1) la instrucción verbal debe adaptarse o ampliarse para facilitar el inicio de la actividad; 2) el/la evaluador/a debe realizar la actividad primero o mostrar cómo se inicia; 3) el/la evaluador/a debe mostrar imágenes o proporcionar pistas escritas para ayudar a la persona a comenzar; 4) los objetos deben ser entregados a la persona evaluada para poder iniciar la actividad
2 puntos	Se da al menos uno de los siguientes supuestos: 1) el/la evaluador/a debe comenzar la actividad junto con el paciente; 2) la actividad debe adaptarse para que la persona pueda comenzarla de manera adecuada
3 puntos	La persona no puede iniciar la actividad y es el/la evaluador/a quién tiene que hacerse cargo
**Ejecución:** capacidad para ejecutar correctamente las acciones práxicas de las actividades en términos de aspectos temporales, espaciales y de contenido de la acción	
0 puntos	No se observan dificultades ejecutando las acciones práxicas
1 punto	Se da al menos uno de los siguientes supuestos: 1) la persona necesita orientación verbal para realizar las acciones; 2) la orientación verbal debe combinarse con gestos, mímica o entonación para realizar las acciones práxicas de la actividad
2 puntos	La persona requiere una orientación física para realizar las acciones práxicas de la actividad
3 puntos	El/la evaluador/a debe hacerse cargo de la actividad
**Control:** capacidad de monitorear la calidad de las acciones práxicas durante la ejecución de movimientos y la calidad de los resultados obtenidos después de la actividad	
0 puntos	No se observan problemas, la persona no requiere retroalimentación
1 punto	Se da al menos uno de los siguientes supuestos: 1) la persona necesita retroalimentación verbal sobre el resultado de la actividad; 2) la persona necesita retroalimentación física sobre el resultado de la actividad
2 puntos	Se da al menos uno de los siguientes supuestos: 1) la persona necesita retroalimentación verbal sobre la ejecución de la actividad; 2) la persona necesita retroalimentación física sobre la ejecución de la actividad; 3) la persona requiere el uso de espejos o grabaciones en video que permitan a la persona observar su ejecución
3 puntos	El/la evaluador/a debe corregir la actividad en su totalidad

### Phase II: Psychometric properties of the Spanish version of the ADL Observation Scale

#### Structural validity.

A sample of 180 patients with stroke participated in the present study. The data was considered acceptable for factor analysis based on a p-value <0.01 for Bartlett’s test and a KMO value of 0.927.

**Fig 2:**
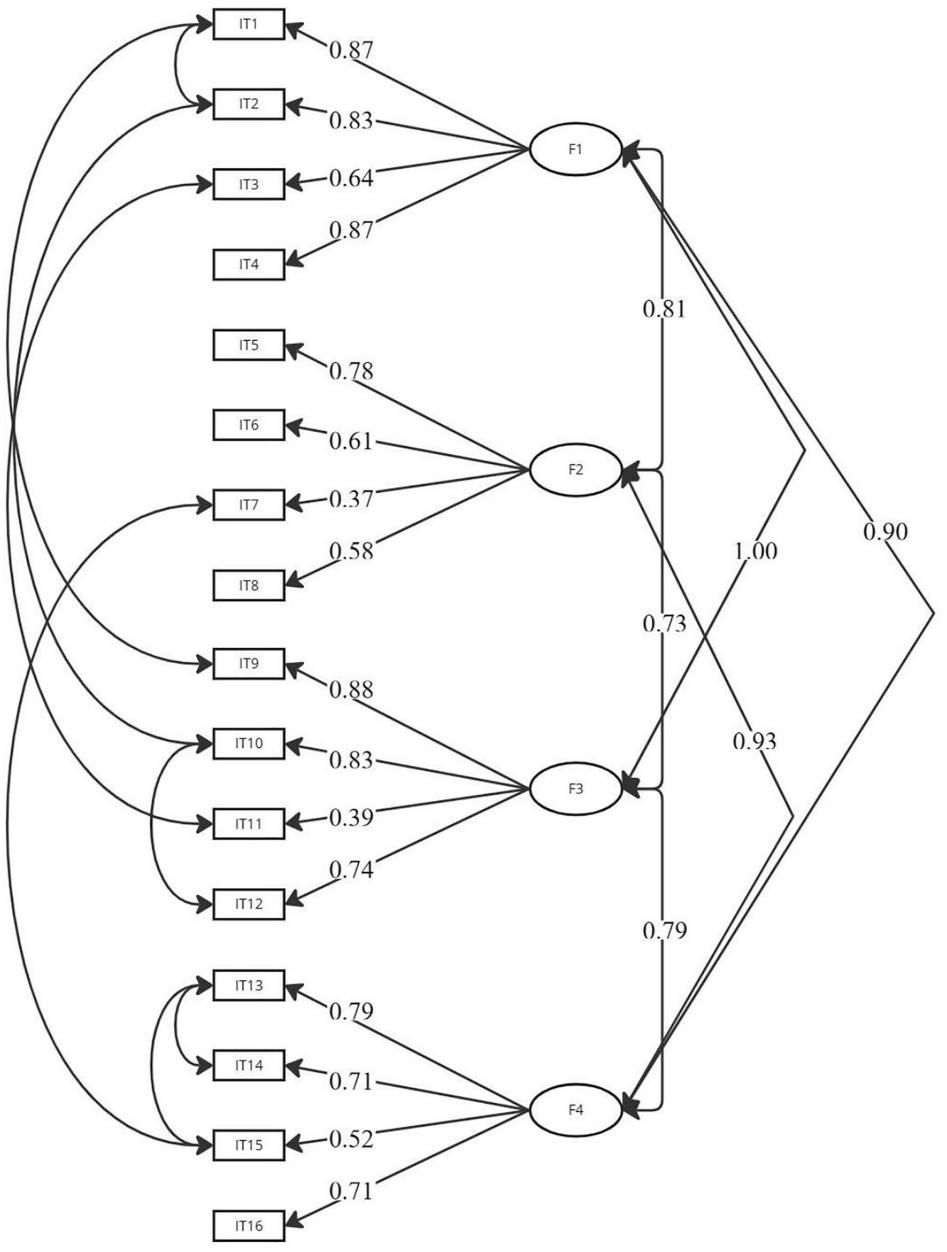
Path diagram of Model 2 of confirmatory factor analysis of the Spanish version of the ADL Observation Scale. F1 = Factor 1 (independence); F2 = Factor 2 (initiation); F3 = Factor 3 (execution); F4 = Factor 4 (control); IT = Item.

Three models were analyzed using CFA. Model 1 (unifactorial structure, CFI = 0.848), Model 2 (including the factors of independence, initiation, execution, and control, CFI = 0.952), showing a high correlation between independence and execution factors, and Model 3 (including initiation, control, and the unified independence-execution factor, CFI = 0.859). The model fit statistics provided by AMOS software showed that the four-factor model (Model 2) has a significantly better fit for the data compared to the other models. Fig 2 shows the path diagram of this four-factor model, and Table 3 presents the fit indices of the three models.

**Table 3:** Fit indices for the confirmatory factor analysis of the Spanish version of the ADL Observation Scale.

Model	*χ* ^*2*^ */df*	*p-value*	RMSEA	90% CI	CFI	TLI	SRMR
1	3.932	<0.001	0.128	0.115, 0.141	0.848	0.824	0.720
2	2.095	<0.001	0.078	0.062, 0.094	0.952	0.934	0.045
3	3.346	<0.001	0.114	0.101, 0.128	0.882	0.859	0.664

Model 1 = Unifactorial structure; Model 2 = Four-factor model with the factors of praxis function (independence, initiation, execution, and control); Model 3 = Three-factor model including initiation, control, and the unified independence-execution factor; *χ*^*2*^*/df* = Chi-square and degrees of freedom; RMSEA = Root Mean Square Error of Approximation; CI = Confidence Interval; CFI = Comparative Fit Index; TLI = Tucker-Lewis Index; SRMR = Standardized Root Mean Square Residual.

#### Internal consistency.

The Cronbach’s alpha and McDonald’s omega coefficient results indicated good levels of internal consistency except for the initiation factor, which was slightly below the threshold in both age groups. Table 4 shows the results of the internal consistency coefficients (Cronbach’s alpha values for Spanish and English versions), and the item-factor correlation ranges.

**Table 4:** Internal consistency across age groups and language versions of the ADL Observation Scale.

Factor	Spanish version (N = 180)					English version (N = 36)
	Cronbach’s alpha [95% CI]		McDonald’s omega coefficients [95% CI]		Item-factor correlations (range)	Cronbach’s alpha [95% CI]
	≤65 years	>65 years	≤65 years	>65 years		
1	0.893 [0.854, 0.924]	0.886 [0.839, 0.921]	0.915 [0.882, 0.936]	0.906 [0.865, 0.933]	0.64-0.87	0.79
2	0.678 [0.559, 0.771]	0.641 [0.495, 0.753]	0.756 [0.484, 0.862]	0.674 [0.476, 0.805]	0.37-0.78	0.81
3	0.900 [0.863, 0.929]	0.855 [0.796, 0.900]	0.906 [0.861, 0.931]	0.864 [0.801, 0.902]	0.39-0.88	0.77
4	0.769 [0.684, 0.835]	0.775 [0.683, 0.845]	0.797 [0.677, 0.855]	0.802 [0.686, 0.871]	0.52-0.79	0.81

Factor 1 = independence; Factor 2 = initiation; Factor 3 = execution; Factor 4 = control.

#### Identification of cut-off points and diagnostic accuracy.

Cut-off points and their accuracy were estimated for each of the four factors composing the ADL Observation Scale and adjusted for age. ROC curve analysis showed AUC values ranging from 0.687 to 0.932. These values indicated that the ADL Observation Scale has a discrimination accuracy greater than 69%. The optimal cut-off points, identified using the Youden index, ranged between ≥1 and ≥4 points for each factor. Based on these points, for participants under 65 years old, the optimal balance between sensitivity and specificity was found in the “execution” factor, with a sensitivity of 0.861 [0.725, 0.948] and a specificity of 0.806 [0.697, 0.891]. For participants over 65 years old, the optimal balance was achieved in the “independence” factor, showing a sensitivity of 0.829 [0.683, 0.928] and a specificity of 0.872 [0.758, 0.947]. Table 5 summarizes the results of the cut-off points, sensitivity, and specificity analysis.

**Table 5:** Characteristics of the ROC analysis of the Spanish version of the ADL Observation Scale: area under the curve, Youden index, cut-off points, sensitivity, specificity, and confidence interval for each factor (adjusted for age).

≤65 years old (*n* = 98)											
ADL Observation Scale factors	AUC	95% CI AUC		Youden index	Cut-off points	Sensitivity	95% CI Sensitivity		Specificity	95% CI Specificity	
		LB	UB				LB	UB		LB	UB
Independence	0.894	0.823	0.966	0.637	≥3	0.750	0.594	0.871	0.887	0.793	0.950
Initiation	0.687	0.570	0.804	0.368	≥1	0.417	0.266	0.579	0.952	0.879	0.988
Execution	0.880	0.804	0.956	0.668	≥2	0.861	0.725	0.948	0.806	0.697	0.891
Control	0.821	0.723	0.918	0.614	≥1	0.694	0.534	0.828	0.919	0.835	0.970
**>65 years old (*n* = 82)**											
**ADL Observation Scale factors**	**AUC**	**95%** **CI AUC**		**Youden index**	**Cut-off points**	**Sensitivity**	**95% CI Sensitivity**		**Specificity**	**95% CI Specificity**	
		**LB**	**UB**				**LB**	**UB**		**LB**	**UB**
Independence	0.932	0.877	0.987	0.701	≥4	0.829	0.683	0.928	0.872	0.758	0.947
Initiation	0.787	0.682	0.891	0.559	≥1	0.771	0.616	0.888	0.787	0.657	0.887
Execution	0.884	0.809	0.960	0.687	≥3	0.857	0.718	0.946	0.830	0.706	0.918
Control	0.820	0.720	0.920	0.551	≥2	0.657	0.493	0.799	0.894	0.785	0.960

ADL = Activities of Daily Living; ROC = Receiver Operating Characteristic, AUC = Area Under the Curve; CI = Confidence Interval; LB = Lower Bound; UB = Upper bound.

The positive predictive values for the ADL Observation Scale ranged from 0.721 [0.675, 0.937] to 0.833 [0.623, 0.956], indicating that the scale effectively identifies more than 72% of patients with praxis deficits regardless of age. On the other hand, the negative predictive value ranged from 0.738 [0.635, 0.825] to 0.909 [0.815, 0.966], which indicates that the ADL Observation Scale can identify correctly more than 74% of individuals who do not exhibit praxis deficits. The remaining results are presented in Table 6.

**Table 6:** Predictive values and confidence interval (adjusted for age) of the Spanish ADL Observation Scale.

ADL Observation Scale factors						
≤65 years old (*n* = 98)						
	PPV	95% CI PPV		NPV	95% CI NPV	
		Lower bound	Upper bound		Lower bound	Upper bound
Independence	0.794	0.640	0.906	0.859	0.761	0.930
Initiation	0.833	0.623	0.956	0.738	0.635	0.825
Execution	0.721	0.577	0.840	0.909	0.815	0.966
Control	0.833	0.675	0.937	0.838	0.739	0.912
**>65 years old (*n* = 82)**						
Independence	0.829	0.683	0.928	0.872	0.758	0.947
Initiation	0.730	0.574	0.854	0.822	0.694	0.915
Execution	0.789	0.643	0.898	0.886	0.771	0.958
Control	0.821	0.655	0.932	0.778	0.656	0.874

ADL = Activities of Daily Living; PPV = Positive Predictive Value; CI = Confidence Interval; NPV = Negative Predictive Value.

## Discussion

This study aimed to cross-cultural adapt and assess the psychometric properties of the ADL Observation Scale in a sample of Spanish-speaking patients with stroke. The findings showed that the Spanish version is conceptually, semantically, and operationally equivalent to the original scale. The assessment of structural validity revealed an adequate four-factor structure, with high internal consistency for these factors, except for the initiation factor, which presented a value slightly below the recommended thresholds. Optimal cut-off points were identified for each factor, showing high accuracy in distinguishing specific praxis deficits during the performance of ADL. The predictive values also supported the scale’s ability to accurately classify patients with praxis deficits.

In the cross-cultural adaptation, special attention was paid to ensuring equivalence with the original version and cultural relevance for the target population, as in other previously validated instruments evaluating ULA [54]. To improve the standardization of the scale in clinical and research settings, all parts were described in detail, including the ADLs used as items to assess praxis function, the underlying factors, and scoring methods. In this line, an application manual was developed to let professionals understand what is expected and thus facilitate the administration of the scale [29]. All these adaptations contributed to improving the precision of the scale, supporting a more consistent and replicable evaluation of praxis abilities across diverse populations [55].

The four-factor structure of the ADL Observation Scale reflects the multidimensionality of praxis functioning in the performance of daily life activities, including independence in activity actions/steps, actions/steps initiation, actions/steps execution, and actions/steps control. This model allows for differentiated evaluation of praxis deficits, which is essential for guiding rehabilitation interventions to patients’ particular needs. While the independence and execution factors showed high correlations, the three-factor model did not provide a better fit to the data. Moreover, previous literature and the theoretical rationale of the scale support maintaining these four factors as distinct components, as each captures different aspects of praxis function [6,9].

The internal consistency of the ADL Observation Scale was adequate regardless of the age of the patients, except for the initiation factor. This factor showed a value slightly below the recommended threshold for individuals below and above 65 years old. To provide more precise support to the internal consistency findings, McDonald’s omega coefficient was also calculated. Internal consistency of the initiation factor contrasts with the original version of the scale, which reported higher internal consistency. One possible explanation for this finding is the limited response variability since this study included a sample of patients with mild to moderate stroke. In clinical terms, this result suggests that in a sample with similar characteristics —patients with mild to moderate stroke— it may not be necessary to evaluate the initiation factor, as they do not appear to exhibit alterations in this aspect of praxis function. Nevertheless, since praxis function in daily life has been shown to be composed of four factors, this structure should be retained. Future studies are needed to support the reliability of this specific factor in more heterogeneous samples, including patients with severe stroke.

The validation of the ADL Observation Scale provides a valuable clinical perspective for adapting rehabilitation strategies. In this study, cut-off points and diagnostic accuracy indices (sensitivity, specificity, and predictive values) were calculated for each factor and adjusted for age, offering clinicians precise criteria to identify praxis deficits during performance of ADL. If patients score equal to or above the established cut-off point on a factor, this factor of praxis function can be considered altered. For example, a score ≥3 on the independence factor may indicate difficulties in functional autonomy and the need for interventions aimed at improving praxis function related to independence in daily activities, whereas a score ≥2 on the execution factor would suggest deficits in the kinematic organization of movement during ADL execution. Deficits in this latter dimension, for example, should be addressed by focusing on reducing spatial, temporal, and postural errors during functional movements. This information allows clinicians not only to detect the presence or absence of ULA but also to specify how ULA is affecting daily life, and which specific praxis components require targeted intervention. Consequently, rehabilitation can be personalized and resources optimized, reducing the delays in the detection and management of these functional deficits. On the other hand, it is important to acknowledge that other cognitive domains, such as visuospatial abilities, attention or memory [56], as well as psychosocial factors, like self-efficacy or spirituality [57,58], may act as mediators of performance in movements, gestures, actions, tasks and activities among patients with stroke.

### Limitations of the study

The present study should be interpreted considering some limitations. First, the sample was recruited from a province in Spain (Granada), which may limit the representativeness across diverse Spanish-speaking countries. Second, some degree of overfitting was necessary to apply to the CFA analysis, but the model fit of Model 2 was supported statistically and conceptually. Third, the confidence intervals observed for the positive and negative predictive values may reduce the precision of these estimates. The interpretation of these results should be approached with caution. Fourth, the temporal stability and the concurrent validity of the ADL Observation Scale were not assessed. Future studies should also address these analyses and recruit more diverse samples from multiple provinces and Spanish-speaking countries to enhance the generalizability of the results. Also, research should consider assessing these aspects to better understand their potential relationship to praxis function in daily life after stroke.

## Conclusions

This is the first study that cross-cultural adapts and evaluates the psychometric properties of the ADL Observation Scale in Spanish, and the findings show that it is a valid instrument for evaluating the levels of praxis functioning in the performance of daily life activities. Cross-cultural adaptation of the scale guarantees equivalence with the original scale. Structural validity assessment supports a four-factor structure based on the four factors of praxis function. The ADL Observation Scale is also a reliable instrument with optimal cut-off points identified for each of the four factors and adjusted for age, which improves the accuracy of the scale. Furthermore, the scale presents high sensitivity and specificity in identifying patients with praxis deficits. The positive and negative predictive values confirm the precision of the ADL Observation Scale to correctly classify the patients who had deficits in praxis.
